# Hydrazine Radiolysis by Gamma-Ray in the N_2_H_4_–Cu^+^–HNO_3_ System

**DOI:** 10.3390/ijms22147376

**Published:** 2021-07-09

**Authors:** Naon Chang, Huijun Won, Sangyoon Park, Heechul Eun, Seonbyeong Kim, Bumkyung Seo, Yongsoo Kim

**Affiliations:** 1 Department of Nuclear Engineering, Hanyang University, 222 Wangsimri-ro, Seongdong-gu, Seoul 04763, Korea; nochang@hanayang.ac.kr; 2 Decommissioning Technology Research Division, Korea Atomic Energy Research Institute, Daedeok-daero 989-111, Yuseong-gu, Daejeon 34057, Korea; nsypark@kaeri.re.kr (S.P.); ehc2004@kaeri.re.kr (H.E.); sbkim@kaeri.re.kr (S.K.); bumja@kaeri.re.kr (B.S.); 3 Quantum Energy Chemical Engineering, University of Science and Technology, Gajeong-ro 217, Yuseong-gu, Daejeon 34113, Korea

**Keywords:** radiolysis, γ-ray, irradiation, hydrazine, copper ion, nitrate ion

## Abstract

Radiolysis of chemical agents occurs during the decontamination of nuclear power plants. The γ-ray irradiation tests of the N_2_H_4_–Cu^+^–HNO_3_ solution, a decontamination agent, were performed to investigate the effect of Cu^+^ ion and HNO_3_ on N_2_H_4_ decomposition using a Co-60 high-dose irradiator. After the irradiation, the residues of N_2_H_4_ decomposition were analyzed by Ultraviolet-visible (UV) spectroscopy. NH_4_^+^ ions generated from N_2_H_4_ radiolysis were analyzed by ion chromatography. Based on the results, the decomposition mechanism of N_2_H_4_ in the N_2_H_4_–Cu^+^–HNO_3_ solution under γ-ray irradiation condition was derived. Cu^+^ ions form Cu^+^N_2_H_4_ complexes with N_2_H_4_, and then N_2_H_4_ is decomposed into intermediates. H^+^ ions and H^●^ radicals generated from the reaction between H^+^ ion and e_aq_^−^ increased the N_2_H_4_ decomposition reaction. NO_3_^−^ ions promoted the N_2_H_4_ decomposition by providing additional reaction paths: (1) the reaction between NO_3_^−^ ions and N_2_H_4_^●+^, and (2) the reaction between NO^●^ radical, which is the radiolysis product of NO_3_^−^ ion, and N_2_H_5_^+^. Finally, the radiolytic decomposition mechanism of N_2_H_4_ obtained in the N_2_H_4_–Cu^+^–HNO_3_ was schematically suggested.

## 1. Introduction

Hydrazine (N_2_H_4_) is commercially used to produce plastics, medicines, and textile dyes and to reduce the corrosion of a boiler in a thermal power plant [1,2]. In the nuclear field, N_2_H_4_ is added to the primary feed water to maintain the hydrogen concentration and to remove dissolved oxygen [3]. In addition, N_2_H_4_ can be applied as a chemical decontamination solution to remove radioactive nuclides in an oxide layer of a primary system in the nuclear power plant [4]. The decontamination solution containing N_2_H_4_ is developed to reduce the damage of the base metal and the secondary radioactive wastes compared with the decontamination using organic acid [5,6]. They are composed of N_2_H_4_ and inorganic acids such as HNO_3_ and H_2_SO_4_ [5,6,7]. Furthermore, the metal ions can be added into the decontamination solution containing N_2_H_4_ for improving the decontamination performance [8]. For this reason, a N_2_H_4_–Cu^+^–HNO_3_ decontamination solution was suggested as the effective chemical decontamination solution [7]. The decontamination solution, however, can be decomposed under the high radiation field [9]. The radiolysis of the decontamination solution occurs by radionuclides in the primary system, such as Co-60 and Co-58, during the application. The decomposition of major compositions of the decontamination solution affects the decontamination performance. Therefore, it is necessary to analyze the radiolysis of N_2_H_4_, which is the major component of the N_2_H_4_–Cu^+^–HNO_3_ decontamination solution, during irradiation.

In this regard, a number of research studies concerning decomposition of N_2_H_4_ solution have been carried out. It is known that the decomposition of N_2_H_4_ under irradiation conditions can occur through a reaction with radiolysis products of water. Various radicals and products, such as e_aq_^−^, ^•^OH, and H_3_O^+^, are generated by the radiolysis of water [10]. The radiolysis products of water as represented in Equation (1) [11].

(1) $$\begin{array}{c} H_{2}O\overset{\gamma -\mathrm{radiation}}{\to }0.27e_{aq}{}^{-},\;0.06\;H^{•},\;0.26^{•}OH,\;0.045H_{2},\;0.08\;H_{2}O_{2},\;0.27H_{3}O^{+} \end{array}$$

The decomposition reaction mechanism of N_2_H_4_ in the aqueous solution by γ-ray irradiation can be found in the study of Buxton et al. [12]. They reported that radicals such as N_2_H_4_^●+^, NH_2_^●^ and N_2_H_3_^●^, generated from N_2_H_4_, are decomposed into N_2_ and NH_3_. It is also shown that the NH_4_^+^ ion was produced by a reaction between H^●^ radical and N_2_H_5_^+^. Garaix et al. studied the decay mechanism of the NO_3_^●^ radical generated by the radiolysis of NO_3_^−^ ions in the N_2_H_4_ solution using an electron beam [13]. They concluded that N_2_H_4_ exists mainly as N_2_H_5_^+^ or N_2_H_6_^2+^ in the acidic solution, both of which cause rapid consumption of NO_3_^●^ radicals. Motooka et al. also reported that the deoxygenation reaction with radiolysis of N_2_H_4_ can be suppressed by salt in the water in the γ-radiation field [14]. In this way, the molecular structure of N_2_H_4_ after radiolysis and the decomposition mechanism of the N_2_H_4_ depend on the composition of the solution. However, there are few research studies about the N_2_H_4_ decomposition reaction in N_2_H_4_–Cu^+^–HNO_3_ solution. Therefore, it is necessary to study the decomposition reaction mechanism of the N_2_H_4_ in N_2_H_4_–Cu^+^–HNO_3_ solution during γ-ray irradiation to simulate the decontamination condition.

In this study, we evaluated the effects of Cu^+^ ions and HNO_3_ on N_2_H_4_ decomposition under the γ-radiation field. The study was performed by analyzing the concentration of remaining N_2_H_4_ and the concentration of the products in N_2_H_4_–Cu^+^–HNO_3_ solution after the irradiation. The decomposition mechanism of N_2_H_4_ in the solution containing Cu^+^ ions and HNO_3_ was also suggested.

## 2. Theoretical Background

### 2.1. Radiolysis of Hydrazine in Acidic Solution

Hydrazine generally exists in the form of N_2_H_5_^+^ by its reaction with H^+^ ions in an acidic solution, as given in Equation (2) [13,15,16]. At pH 1, N_2_H_6_^2+^ coexists with N_2_H_5_^+^ through the reaction shown in Equation (3) [13,15,17]. Therefore, N_2_H_4_ exists in the forms of N_2_H_5_^+^ and N_2_H_6_^2+^ in the N_2_H_4_–HNO_3_ system before irradiation.

N_2_H_4_ + H^+^ 2194 N_2_H_5_^+^, p*K* ≈ 7.9 (2)

N_2_H_5_^+^ + H^+^ ↔ N_2_H_6_^2+^, p*K* ≈ −1 (3)

In an acidic solution, the same as the condition of this study, e_aq_^−^, H^●^, OH^●^, and H_2_O_2_ are generated as products after water radiolysis. It is possible that e_aq_^−^ reacts with H^+^ ions in the acidic solution and generates H^●^ as in the following Equation (4) [18]. The reactions between the water radiolysis products and the chemical species of N_2_H_4_ lead to the decomposition of N_2_H_4._

e_aq_^−^ + H^+^ → H^●^, *k* = 2.2 × 10^10^ M^−1^s^−1^ (4)

The principal decomposition reactions and rate constants of the chemical species of N_2_H_4_ in the irradiation condition are listed in Equations (5)–(23). As shown in Equation (5), N_2_H_6_^2+^ reacts with OH^●^ and produces N_2,_ the end product of N_2_H_4_ decomposition at pH 1 [19].

N_2_H_6_^2+^ + 4OH^●^ → N_2_ + 4H_2_O + 2H^+^ (5)

In addition, N_2_H_5_^+^ is the main species form of N_2_H_4_ in the acidic solution. N_2_H_5_^+^ reacts with the radiolysis products of water such as e_aq_^−^, H^●^, or OH^●^ as shown in Equations (6)–(9) [12,20]. NH_4_^+^ ion, one of the end products of N_2_H_4_ decomposition, is produced by the reaction between N_2_H_5_^+^ and H^●^, as indicated in Equation (7). The intermediates of N_2_H_5_^+^ decomposition, N_2_H_4_, NH_2_^●^_,_ and N_2_H_4_^●+^, are generated by the reactions in Equations (6), (8) and (9). These intermediates cause the consecutive decomposition reactions of N_2_H_4_. In particular, N_2_H_4_ can also be hydrolyzed into N_2_H_5_^+^ and N_2_H_6_^2+^ as shown in Equations (2) and (3).

The consecutive decomposition reactions of N_2_H_4_ with OH^●^, N_2_H_4_^●+^, and H_2_O_2_ are listed in Equations (10)–(12) [12,20,21]. N_4_H_8_^+^ is formed by the reaction between N_2_H_4_ and N_2_H_4_^●+^, as indicated in Equation (10). ^●^N_2_H_3_ is generated by the reaction between N_2_H_4_ and OH^●^, as shown in Equation (11). The intermediates, N_4_H_8_^+^ and ^●^N_2_H_3_, participate in the other consecutive decomposition reactions of N_2_H_4_. However, N_2_ is produced as the end product by the reaction between N_2_H_4_ and H_2_O_2_ (Equation (12)).

NH_2_^●^ generated by the reaction in Equation (7) causes the reactions with N_2_H_5_^+^ or N_2_H_4_, as represented in Equations (13) and (14) [12]. N_2_H_4_^●+^, ^●^N_2_H_3_, and NH_3_ are formed after the reactions shown in Equations (13) and (14). Among these products, N_2_H_4_^●+^ and ^●^N_2_H_3_ cause the consecutive reactions because they are the reactive intermediates.

N_2_H_4_^●+^ produced by Equations (8), (9) and (13) participate in the consecutive reactions represented in Equations (15) and (16) [12]. As shown in Equation (15), N_4_H_9_^●2+^ is generated after the reaction of N_2_H_4_^●+^ with N_2_H_5_^+^. As indicated in Equation (16), N_4_H_9_^●2+^ reacts with N_2_H_4_^●+^, and the reaction products are N_4_H_8_^2+^ and N_2_H_5_^+^. The N_4_H_8_^2+^ is directly decomposed into N_2_ and NH_3_ in the ratio of 1 to 2. On the other hand, N_2_H_5_^+^ is repeatedly decomposed into other forms, as represented in Equations (6)–(9), (13) and (15).

^●^N_2_H_3_ generated by Equations (11) and (14) is decomposed into various forms, as listed in Equations (17)–(22) [12]. The main end products of ^●^N_2_H_3_ decomposition are N_2_ and NH_3_, as represented in Equations (19) and (20). The main intermediates, N_2_H_4_ and N_2_H_2_, are also generated from the decomposition reaction of ^●^N_2_H_3_, as shown in Equations (17)–(22). N_2_H_4_ is hydrolyzed into N_2_H_5_^+^ and N_2_H_6_^2+^ in the acidic solution or causes consecutive decomposition reactions. N_2_H_2_ reacts with H^●^, and ^●^N_2_H_3_ is produced as shown in Equation (23).

As mentioned above, it is expected that various intermediates are generated during the decomposition of the chemical species of N_2_H_4_. Therefore, the intermediates can affect the reaction with Cu^+^ ions or NO_3_^−^ ions in the N_2_H_4_–Cu^+^–HNO_3_ system.

N_2_H_5_^+^ + e_aq_^−^ → H^●^ + N_2_H_4_, *k* = 1.6 × 10^8^ M^−1^s^−1^ (6)

N_2_H_5_^+^ + H^●^ → NH_2_^●^ + NH_4_^+^, *k* = 1.0 × 10^4^ M^−1^s^−1^ (7)

N_2_H_5_^+^ + H^●^ → H_2_ + N_2_H_4_^●+^, *k* = 1.3 × 10^5^ M^−1^s^−1^ (8)

N_2_H_5_^+^ + OH^●^ → N_2_H_4_^●+^ + H_2_O, *k* = 8.2 × 10^7^ M^−1^s^−1^ (9)

N_2_H_4_ + N_2_H_4_^●+^ ↔ N_4_H_8_^●+^, *k_f_* = 6.0 × 10^7^ M^−1^s^−1^, *k_b_* = 4.0 × 10^5^ M^−1^s^−1^ (10)

N_2_H_4_ + OH^●^ → ^●^N_2_H_3_ + H_2_O, *k* = 5.4 × 10^9^ M^−1^s^−1^ (11)

N_2_H_4_ + 2H_2_O_2_ → N_2_ + 4H_2_O, *k* = 2.4 × 10^8^ M^−1^s^−1^ (12)

NH_2_^●^ + N_2_H_5_^+^ → N_2_H_4_^●+^ + NH_3_, *k* = 1.0 × 10^6^ M^−1^s^−1^ (13)

NH_2_^●^ + N_2_H_4_ → ^●^N_2_H_3_ + NH_3_, *k* = 1.0 × 10^7^ M^−1^s^−1^ (14)

N_2_H_4_^●+^ + N_2_H_5_^+^ ↔ N_4_H_9_^●2+^, *k_f_* = 6.0 × 10^7^ M^−1^s^−1^, *k_b_* = 4.0 × 10^5^ M^−1^s^−1^ (15)

N_2_H_4_^●+^ + N_4_H_9_^●2+^ → N_4_H_8_^2+^ + N_2_H_5_^+^ (N_4_H_8_^2+^ → N_2_ + 2NH_3_), *k* = 4.0 × 10^8^ M^−1^s^−1^ (16)

^●^N_2_H_3_ + e_aq_^−^ + H_2_O → N_2_H_4_ + OH^−^, *k* = 7.0 × 10^9^ M^−1^s^−1^ (17)

^●^N_2_H_3_ + H^●^ → N_2_H_4_, *k* = 7.0 × 10^9^ M^−1^s^−1^ (18)

^●^N_2_H_3_ + N_4_H_8_^●+^ → N_2_H_4_ + N_4_H_7_^+^ (N_4_H_7_^+^ → N_2_ + 2NH_3_), *k* = 1.0 × 10^9^ M^−1^s^−1^ (19)

^●^N_2_H_3_ + N_4_H_9_^●2+^ → N_2_H_4_ + N_4_H_8_^2+^ (N_4_H_8_^2+^ → N_2_ + 2NH_3_), *k* = 1.0 × 10^9^ M^−1^s^−1^ (20)

^●^N_2_H_3_ + N_2_H_4_^●+^ → N_2_H_2_ + N_2_H_5_^+^, *k* = 7.0 × 10^8^ M^−1^s^−1^ (21)

^●^N_2_H_3_ + ^●^N_2_H_3_ → N_2_H_2_ + N_2_H_4_, *k* = 6.0 × 10^8^ M^−1^s^−1^ (22)

N_2_H_2_ + H^●^ → ^●^N_2_H_3_, *k* = 3.0 × 10^9^ M^−1^s^−1^ (23)

### 2.2. Change of Copper Species during Irradiation

Copper ions in the solution would cause the decomposition of N_2_H_4_ during the irradiation. The redox reactions mainly occur between copper ions and radiolysis products of water such as e_aq_^−^, H^●^, and OH^●^, as listed in Equations (24)–(28) [22,23,24,25]. The equations show that copper ions coexist in the forms of Cu^0^, Cu^+^ ions and Cu^2+^ ions regardless of initial chemical species. In addition, Fenton reaction occurs in an acidic condition, as represented in Equation (29) [26,27]. The above reactions can affect the decomposition of N_2_H_4_ in the N_2_H_4_–Cu^+^–HNO_3_ system.

Cu^+^ + e_aq_^−^ → Cu^0^ (24)

Cu^+^ + H^●^ → Cu^0^ + H^+^, *k* = 5 × 10^9^ M^−1^s^−1^ (25)

Cu^+^ + OH^●^ → Cu^2+^ + OH^−^, *k* = (2±1) × 10^10^ M^−1^s^−1^ (26)

Cu^2+^ + e_aq_^−^ → Cu^+^, *k* = 3.5 × 10^10^ M^−1^s^−1^ (27)

Cu^2+^ + H^●^ → Cu^+^ + H^+^, *k* < 1.0 × 10^6^ M^−1^s^−1^ (28)

H_2_O_2_ + Cu^+^ + H^+^ → OH^●^ + H_2_O + Cu^2+^ (29)

### 2.3. Radiolysis of Nitrate Ion

The principal reactions of NO_3_^−^ ions during the irradiation are listed in Equations (30)–(38) [13,28,29,30,31,32,33]. The reactions can be classified by direct and indirect decomposition reactions. As shown in Equation (30), the NO_3_^−^ ion is directly changed into NO_3_^●^ and electron due to the γ-ray irradiation [13,28]. The NO_3_^−^ ion is also changed into NO_3_^2−^ ion or NO_2_^●^ through the reactions with e_aq_^−^ or H^●^, as can be seen in Equations (31) and (32) [29,30]. The NO_3_^2−●^ reduces into NO_2_^●^ in the water, as represented in Equation (33) [29,31]. During irradiation, NO_2_^●^ reacts with the radiolysis products of water such as e_aq_^−^, H^●^, and OH^●^, and H^+^, NO_2_^−^ ion, and NO_3_^−^ ions are produced as listed in Equations (34)–(36) [32]. On the other hand, NO_2_^●^ reacts with water and generates NO_2_^−^ and NO_3_^−^ ions, as shown in Equation (37) [13,33]. As represented in Equation (38), NO_2_^−^ ions generated from the reaction in Equations (34), (35) and (37) are changed into NO_3_^−^ ions and NO_2_^●^ through the reaction with NO_3_^●^ [13,33]. The generated NO_2_^−^ ions are directly consumed, and NO_2_^●^ and NO_3_^−^ ions are regenerated by the reaction indicated in Equation (38). As mentioned above, NO_3_^−^ ions and their radicals generated from the radiolysis of NO_3_^−^ can also participate in the decomposition reaction of N_2_H_4_ in the N_2_H_4_–Cu^+^–HNO_3_ system.

(30) $$\begin{array}{c} NO_{3}{}^{-}\overset{\mathrm{radiation}}{\to }NO_{3}{}^{-}*\to NO_{3}{}^{●}+e^{-} \end{array}$$

NO_3_^−^ + e_aq_^−^ → NO_3_^2−●^, *k* = 9.7 × 10^9^ M^−1^s^−1^ (31)

NO_3_^−^ + H^●^ → NO_2_^●^ + OH^−^, *k* = 1.0 × 10^7^ M^−1^s^−1^ (32)

NO_3_^2−●^ + H_2_O → NO_2_^●^ + 2OH^−^, *k* = 1.0 × 10^3^ M^−1^s^−1^ (33)

NO_2_^●^ + e_aq_^−^ → NO_2_^−^, *k* = 1.0 × 10^10^ M^−1^s^−1^ (34)

NO_2_^●^ + H^●^ → H^+^ + NO_2_^−^, *k* = 1.0 × 10^9^ M^−1^s^−1^ (35)

NO_2_^●^ + ^●^OH → H^+^ + NO_3_^−^, *k* = 1.0 × 10^10^ M^−1^s^−1^ (36)

2NO_2_^●^ + H_2_O → NO_2_^−^ + NO_3_^−^ + 2H^+^, *k* = 4.5 × 10^8^ M^−1^s^−1^ (37)

NO_3_^●^ + NO_2_^−^ → NO_2_^●^ + NO_3_^−^, *k* = 4.4 × 10^9^ M^−1^s^−1^ (38)

## 3. Results

### 3.1. Effect of Copper Ions on Hydrazine Decomposition

**Figure 1:**
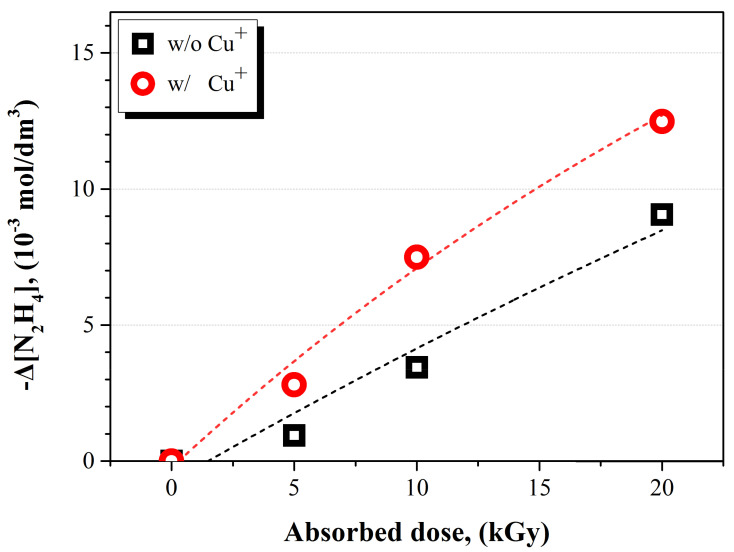
Dependence of N_2_H_4_ decomposition on Cu^+^ ions after γ-irradiation on the solution of which [N_2_H_4_]_0_ = 50 × 10^−^^3^ mol dm^−^^3^ at pH 3.

In order to investigate the effect of copper ions on the N_2_H_4_ decomposition, γ-ray was irradiated to the N_2_H_4_–Cu^+^–HNO_3_ solution and N_2_H_4_–HNO_3_ solution at pH 3. The absorbed dose was varied from 0 to 20 kGy, and the [N_2_H_4_]_0_ in the solutions was equal to 50 × [N2H4]0 in the solutions was equal to 50 × 10^−3^ mol dm^−3^. The pH of the solution was adjusted to 3using HNO_3_. Figure 1 shows the change in the concentration of N_2_H_4_ as a result of the γ-irradiation. The decomposed portion of N_2_H_4_ increased with the increase in the absorbed dose regardless of the presence of the Cu^+^ ions. This result indicates that the amount of radiolysis products of water participating in the N_2_H_4_ decomposition was enhanced when the absorbed dose was increased. At the same absorbed dose, the decomposed portion of N_2_H_4_ was higher when the copper ions existed than that when the copper ions were absent, as indicated in Figure 1. In particular, 12.48 × 10^−3^ mol dm^−3^ of N_2_H_4_ in the solution containing Cu^+^ ions was decomposed after the 20 kGy of γ-irradiation. When the Cu^+^ ions were absent in the solution, 9.05 × 10^−3^ mol dm^−3^ of N_2_H_4_ was decomposed. Moreover, the G-values for the N_2_H_4_ decomposition were calculated for 20 kGy of absorbed dose and listed in Table 1. G(–N_2_H_4_), for the solution containing Cu^+^ ions, was higher than that of the solution not containing Cu^+^ ions.

There are several explanations for the effect of copper ions on the decomposition of N_2_H_4_: (1) a catalyzed reaction of H_2_O_2_ occurs [34], (2) copper ions lower the energy barrier of N-H bonds cleavage in the gas phase [35], and (3) the formation of Cu^+^N_2_H_4_ occurs [36,37]. The experimental condition of Zhong and Lim is similar to that of this study [36,37]. They suggested that Cu^+^N_2_H_4_ complex acts as a scavenger and it increases the decomposition reaction of N_2_H_4_. The predicted mechanism is given in Equations (39)–(41). N_2_H_4_ reacts with Cu^+^ ion and forms the Cu^+^N_2_H_4_ complex, as shown in Equation (39). This complex can react with H_2_O_2_ and produces N_2_H_2_, as indicated in Equation (40). The reaction between the Cu^+^N_2_H_4_ complex and N_2_H_2_ causes the generation of Cu(^●^N_2_H_3_)_2_ that decomposes into Cu^+^ ion and ^●^N_2_H_3_, as represented in Equation (41).

Cu^+^ + N_2_H_4_ ↔ Cu^+^N_2_H_4_ (39)

Cu^+^N_2_H_4_ + H_2_O_2_ → Cu^+^ + N_2_H_2_ + 2H_2_O, (slow) (40)

Cu^+^N_2_H_4_ + N_2_H_2_ ↔ Cu^+^(^●^N_2_H_3_)_2, cage_ → Cu^+^ + 2^●^N_2_H_3_, (slow) (41)

N_2_H_4_ was considered to be reproduced as the intermediate during radiolysis in this study; however, the hydrazine was hydrolyzed into N_2_H_5_^+^ or N_2_H_6_^2+^ in acidic conditions. The N_2_H_4_ could form the Cu^+^N_2_H_4_ complex with the Cu^+^ ion through the reaction, as indicated in Equation (39). As can be seen in Equations (40) and (41), it was possible that the Cu^+^N_2_H_4_ complex was separated into Cu^+^ ion, N_2_H_2_, and water after reacting with H_2_O_2_ generated from the radiolysis of water. The N_2_H_2_ could react with H^●^ and generate ^●^N_2_H_3_ according to the Equation (23). Moreover, N_2_H_2_ could also cause a reaction with the Cu^+^N_2_H_4_ complex and form Cu^+^(N_2_H_3_^●^)_2_. Cu^+^(N_2_H_3_^●^)_2_ was directly decomposed into ^●^N_2_H_3_ and Cu^+^ ion following the reaction in Equation (41). ^●^N_2_H_3_ was decomposed into N_2_ or NH_3_ during the γ-ray irradiation, as listed in Equations (19) and (20). The Cu^+^ ion regenerated from the reactions shown in Equations (40) and (41) repeatedly formed the Cu^+^N_2_H_4_ complex and caused the decomposition reaction of N_2_H_4_ again. Therefore, these two reactions offered new decomposition reaction paths of N_2_H_4_ where the Cu^+^ ion acted as the catalyst.

**Figure 2:**
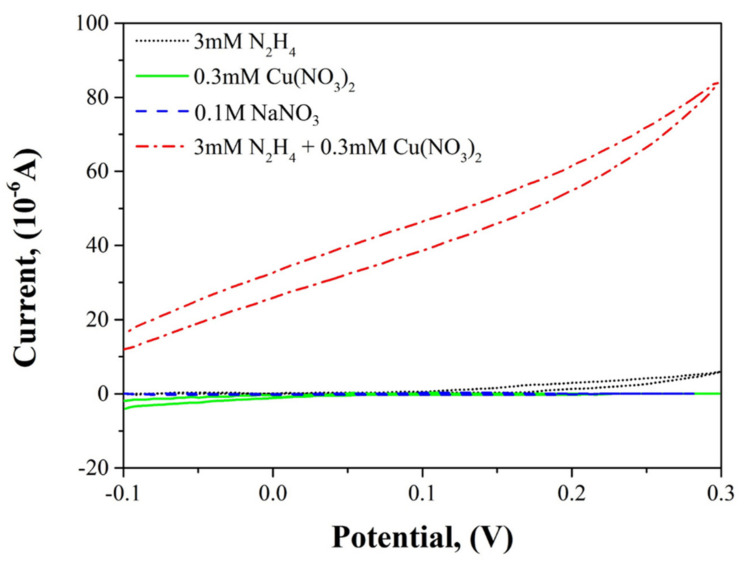
Cyclic voltammograms of 3 mM N_2_H_4_, 0.3 mM Cu(NO_3_)_2_, 0.1 M NaNO_3_, and 3 mM N_2_H_4_ + 0.3 mM Cu(NO_3_)_2_ solutions at scan rate of 20 mV/s. Adapted with permission from [38] Haesik Yang.

The electrochemical characterization, under the same conditions as this experiment, was also performed by Yang et al. [38]. Figure 2 shows the cyclic voltammograms using an ITO (Indium-Tin Oxide) electrode in solutions of 3 mM N_2_H_4_, 0.3 mM Cu(NO_3_)_2_, 0.1 M NaNO_3_, and 3 mM N_2_H_4_ + 0.3 mM Cu(NO_3_)_2_. All the test solutions were adjusted to pH 3 using HNO_3_. The oxidation peak of the N_2_H_4_ near 0.3 V increased significantly with the addition of Cu(NO_3_)_2_. The peak is quite different from that of N_2_H_4_ alone or Cu(NO_3_)_2_ alone. This result implies that Cu^+^ ions make coordination compounds with N_2_H_4_, as listed in reaction Equation (39). Therefore, it is inferred that Cu^+^ ions affect the N_2_H_4_ decomposition by formation of the Cu^+^N_2_H_4_ complex in the N_2_H_4_–Cu^+^–HNO_3_ system.

### 3.2. Effect of HNO_3_ on Hydrazine Decomposition

**Figure 3:**
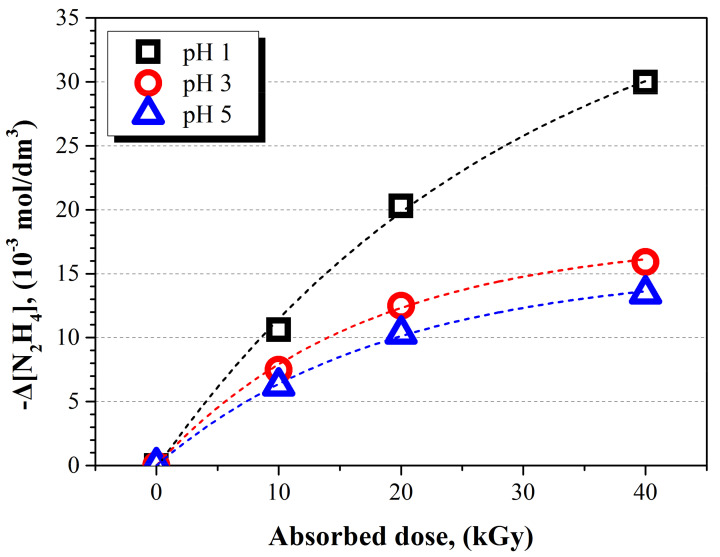
Concentration change of chemical species of N_2_H_4_ according to the absorbed dose under various pHs condition.

HNO_3_ affects the decomposition of N_2_H_4_ in two ways: (1) the effect of H^+^ ions and (2) the effect of NO_3_^−^ ions. In order to investigate the effect of H^+^ ions and NO_3_^−^ ions on N_2_H_4_ decomposition, the concentration of the chemical species of N_2_H_4_ was analyzed according to the pH change under the radiation field. The pH of each solution was adjusted to 1, 3, and 5 by adding HNO_3_, respectively. Every sample solution included 50 × 10^−3^ mol dm^−3^ of N_2_H_4_ and 0.5 × 10^−3^ mol dm^−3^ of Cu^+^ ions. As a result, the concentration of chemical species of N_2_H_4_ decreased with a decreasing pH at the same absorbed dose, as shown in Figure 3. The concentrations of decomposed N_2_H_4_ were 29.96 × 10^−3^ mol dm^−3^ (pH = 1), 15.92 × 10^−3^ mol dm^−3^ (pH = 3), and 13.42 × 10^−3^ mol dm^−3^ (pH = 5) at 40 kGy, respectively. The decomposed portion of N_2_H_4_ significantly increased as the solution’s pH decreased from 3 to 1. At the same time, the G(–N_2_H_4_) at pH 1 was 7.49 × 10^−7^ mol J^−1^ for 40 kGy of absorbed dose, as shown in Table 2. The G(–N_2_H_4_) at pH 3 and 5 were 3.98 × 10^−7^ mol J^−1^ and 3.35 × 10^−7^ mol J^−1^ for 40 kGy of absorbed dose, respectively. Based on this result, it was confirmed that the G-values for the decomposition of N_2_H_4_ increased with the decreasing of the pH.

Firstly, the above results can be explained by the effect of the H^+^ ion on N_2_H_4_ decomposition. As mentioned above, the reaction between the H^+^ ion and e_aq_^−^ caused the generation of H^●^, as represented in Equation (4). The increase in H^●^ promoted the reaction between H^●^ and the intermediates of N_2_H_4_ decomposition, such as N_2_H_5_^+^, N_2_H_3_^●^, and N_2_H_2_, listed in Equations (7), (8), (18) and (23). When the reactions shown in Equations (7), (8) and (23) occurred, NH_4_^+^ ions or the intermediates such as ^●^NH_2_, N_2_H_4_^●+^_,_ and ^●^N_2_H_3_ were produced. As represented in Equation (18), N_2_H_4_ is recovered through the reaction between ^●^N_2_H_3_ and H^●^_._ This N_2_H_4_ could be decomposed after hydrolysis into N_2_H_5_^+^ or be decomposed directly through the reactions listed in Equations (10)–(12). On the other hand, it was possible to form the Cu^+^N_2_H_4_ complex with copper ions and cause a decomposition reaction of N_2_H_4_ using Equations (40) and (41). In particular, N_2_H_4_ and ^●^N_2_H_3_ generated from the reactions shown in Equations (18) and (23) have a high reaction rate, which are 7.0 × 10^9^ M^−1^s^−1^ and 3.0 × 10^9^ M^−1^s^−1^, among the reactions concerned H^●^. The N_2_H_4_ and ^●^N_2_H_3_ are mostly decomposed into N_2_ and NH_3_, as mentioned above.

Secondly, the increase in the decomposition reaction of N_2_H_4_ with the lowering of the pH of the N_2_H_4_–Cu–HNO_3_ solution could also be explained by the effect of the NO_3_^−^ ion. When the NO_3_^−^ ion reacts with NH_4_^●+^, which is the chemical species of N_2_H_4_, N_2_H_2_ and ^●^NO_2_ are produced due to the reaction, as represented in Equation (42) [39]. As listed in Equation (23), the N_2_H_2_ reacts with H^●^, and ^●^N_2_H_3_ is generated. As mentioned above, ^●^N_2_H_3_ normally decomposes into N_2_ and NH_3_, leading to N_2_H_4_ decomposition. ^●^NO_2_ participates in the reaction with radiolysis products of water, and finally NO_3_^−^ is formed by the reaction shown in Equations (34)–(38). On the other hand, NO_3_^●^ generated during the radiolysis of NO_3_^−^ ions also affects N_2_H_4_ decomposition. When NO_3_^●^ reacts with N_2_H_5_^+^, N_2_H_4_^●+^ and HNO_3_ are produced, as shown in Equation (43) [13,39]. N_2_H_4_^●+^ is consecutively decomposed not only by the reaction listed in Equations (15) and (16) but also by the reaction shown in Equation (42). NO_3_^−^ ions recovered from the reaction shown in Equation (43) participate in the decomposition reaction of the chemical species of N_2_H_4_. Therefore, the increase in HNO_3_ in the N_2_H_4_–Cu^+^ solution accelerated the decomposition of N_2_H_4_ by increasing the occurrence of reaction concerned with H^●^ and adding new decomposition reaction paths, including that of the NO_3_^−^ ion.

N_2_H_4_^●+^ + NO_3_^−^ → N_2_H_2_ + H_2_O + ^●^NO_2_, *k* = 2.5 × 10^7^ M^−1^s^−1^ (42)

N_2_H_5_^+^ + NO_3_^●^ → N_2_H_4_^●+^ + NO_3_^−^ + H^+^, *k* = 1.3 × 10^9^ M^−1^s^−1^ (43)

Moreover, the decomposed portion of N_2_H_4_ increased rapidly at pH 1 compared to at pH 3 and 5, when the absorbed doses increased at the rates shown in Figure 3. This was caused by H^●^ and NO_3_^●^ being generated by irradiation. Since the amount of HNO_3_ added at pH 1 was larger than at pH 3 and 5, the amount of H^●^ and NO_3_^●^ produced was larger at pH 1 than at pH 3 and 5. The increase in H^●^ and NO_3_^●^ promoted the decomposition of N_2_H_4_ through the reactions, as mentioned above.

**Figure 4:**
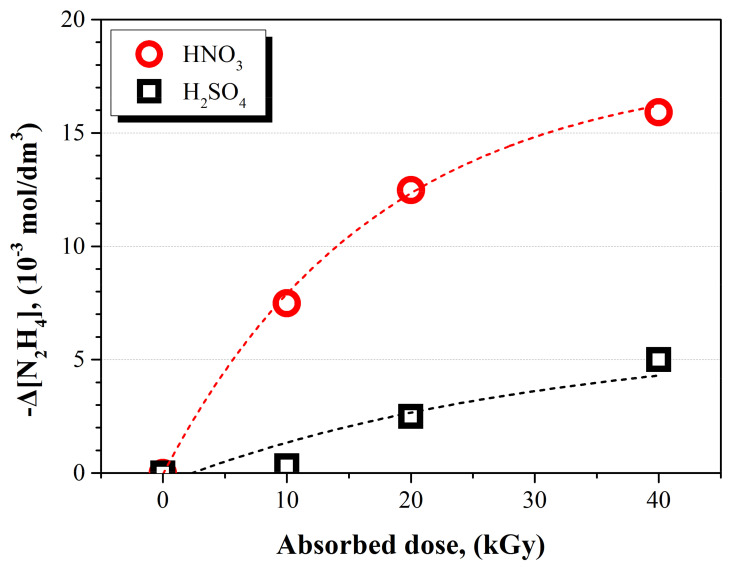
Concentration change of chemical species of N_2_H_4_ according to the absorbed dose under different acid conditions.

In order to investigate the anionic effect, the remaining concentration of N_2_H_4_ in the NO_3_^−^ ion system was compared with that of the SO_4_^2−^ ion system at pH 3. Quantities of 10, 20, and 40 kGy of the absorbed doses were irradiated to each solution. The initial concentration of N_2_H_4_ in each solution was 50 × 10^−3^ mol dm^−3^. As shown in Figure 4, the decomposed concentration of N_2_H_4_ increased when the absorbed dose increased regardless of the type of acid added. At the same absorbed dose, three times higher concentrations of N_2_H_4_ in the NO_3_^−^ ion system were decomposed as compared to the SO_4_^2−^ ion system. As indicated in Table 3, the G-value for the decomposition of N_2_H_4_ at 40 kGy was 3.98 × 10^−7^ mol J^−1^ when the acid added in the solution was HNO_3_. G(–N_2_H_4_) was 1.25 × 10^−7^ mol J^−1^ when the acid injected in the solution was H_2_SO_4_. From these results, we found that the NO_3_^−^ ions facilitated the decomposition of N_2_H_4_ in the solution.

### 3.3. Decomposition Mechanism of Hydrazine in N_2_H_4_–Cu^+^–HNO_3_ System

**Figure 5:**
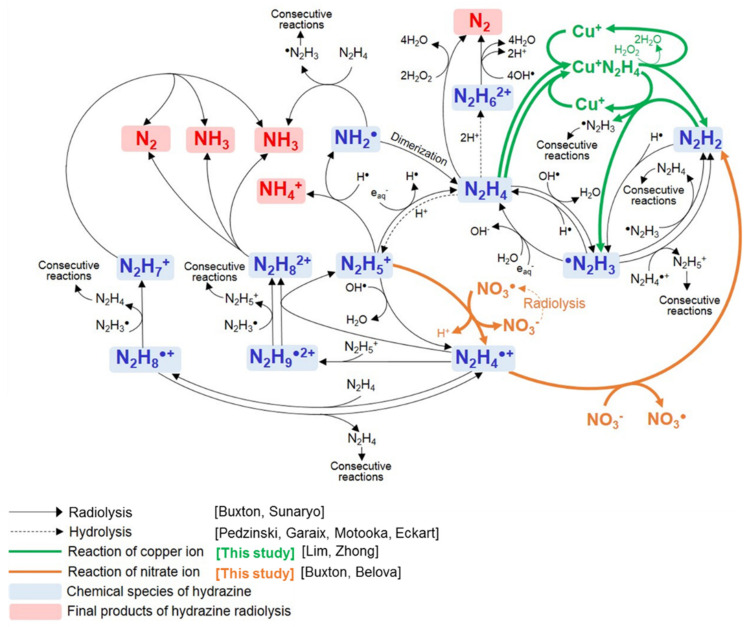
Schematic diagram of radiolysis of N_2_H_4_ in N_2_H_4_–Cu^+^–HNO_3_ system.

The decomposition reactions of the N_2_H_4_ in N_2_H_4_–Cu^+^–HNO_3_ system are schematically shown in Figure 5. N_2_H_4_ in the acidic solution is hydrolyzed and coexists as N_2_H_5_^+^ or N_2_H_6_^2+^. These species are decomposed into intermediates such as N_2_H_4_^●+^, ^●^N_2_H_3_, and NH_2_^●^ under irradiation conditions. N_2_H_4_ can decompose into NH_4_^+^ ion, N_2_, or NH_3_. However, it was verified that the end products were mainly formed with N_2_ or NH_3._ In addition, N_2_H_4_ decomposition was promoted by the influence of the Cu^+^ ion, H^+^ ion, and NO_3_^−^ ion in the N_2_H_4_–Cu^+^–HNO_3_ system, as explained in Section 3.1 and Section 3.2. As represented in green line in Figure 5, Cu^+^ ions form the Cu^+^N_2_H_4_ complex with N_2_H_4_. The Cu^+^N_2_H_4_ complex participates in the reactions, as shown in Equations (40) and (41). The complex decomposes into ^●^N_2_H_3_ and further decomposes into the end products through the consecutive reactions, as listed in Equations (17)–(22). The recovered Cu^+^ ion from the complex repeatedly forms an N_2_H_4_ complex that acts as a catalyst to accelerate the decomposition of N_2_H_4_. H^●^ was produced through the reaction between the H^+^ ion and e_aq_^−^. Therefore, the decomposition reaction of N_2_H_4_ by H^●^ was promoted as the concentration of the H^+^ ion increased. As shown in the orange line in Figure 5, NO_3_^−^ ions or NO_3_^●^ radicals accelerate the N_2_H_4_ decomposition by providing the additional reaction paths to change N_2_H_5_^+^ into N_2_H_4_^●+^. They also cause N_2_H_4_^●+^ to decompose into N_2_H_2._ NO_3_^−^ ion and NO_3_^●^ were regenerated by the radiolysis of NO_2_^●^ and NO_3_^●^, as shown in Equations (34)–(38), and they participated in the N_2_H_4_ radiolysis reaction again. Consequently, N_2_H_4_ decomposition was promoted in the N_2_H_4_–Cu^+^–HNO_3_ system through the mechanism shown in Figure 5.

**Figure 6:**
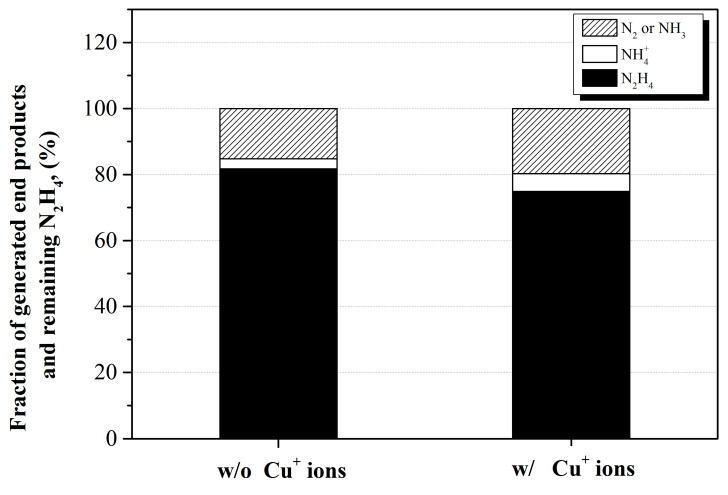
Fraction of radiolysis products and chemical species of N_2_H_4_ in the solution without and with Cu^+^ ions after 20 kGy of γ-ray irradiation.

In order to verify the effect of Cu^+^ ions on the decomposition mechanism of N_2_H_4_ in the N_2_H_4_–HNO_3_ solution, the fraction of N_2_H_4_ and end products were analyzed after irradiation with 20 kGy of the absorbed dose. The initial concentrations of N_2_H_4_ in the solution were 50 × 10^−3^ mol dm^−3^, and the pH of the solution was adjusted to 3. The results were compared with and without Cu^+^ ions in the solution, as represented in Figure 6. Through the above reactions, it was verified that N_2_H_4_ in the N_2_H_4_–Cu^+^–HNO_3_ solution was finally decomposed into N_2_, NH_3_, and NH_4_^+^ ion under an irradiation condition by the reactions with radiolysis products of water or consecutive decomposition reactions. For this reason, the fraction of N_2_ and NH_3_ in the solution after γ-ray irradiation was calculated by subtracting the amount of remaining chemical species of N_2_H_4_ and NH_4_^+^ ions produced after irradiation from the initial amount of N_2_H_4_.

As shown in Figure 6, N_2_H_4_ decomposed into N_2_, NH_3_, and NH_4_^+^ ion. It is well known that most of NH_3_ reacts with H^+^ ions in the acidic solution and exists in the form of NH_4_^+^ [40]. Therefore, it was considered that most of the remaining gas phase end product was composed of N_2_ after irradiation in this study. It was judged that the NH_3_ was converted into NH_4_^+^ ion after the irradiation. When the Cu^+^ ion is present in the N_2_H_4_–HNO_3_ solution, N_2_H_3_^●^ is generated, as indicated in Figure 5 and Equation (41). N_2_H_3_^●^ participated in the reaction, generating N_2_ or NH_3_, as shown in Equations (19) and (20). Therefore, it was confirmed that the fraction of N_2_ and NH_4_^+^, the form of NH_3_ in the acidic condition, increased when the Cu^+^ ions were present in the N_2_H_4_–HNO_3_ solution, as represented in Figure 6.

**Figure 7:**
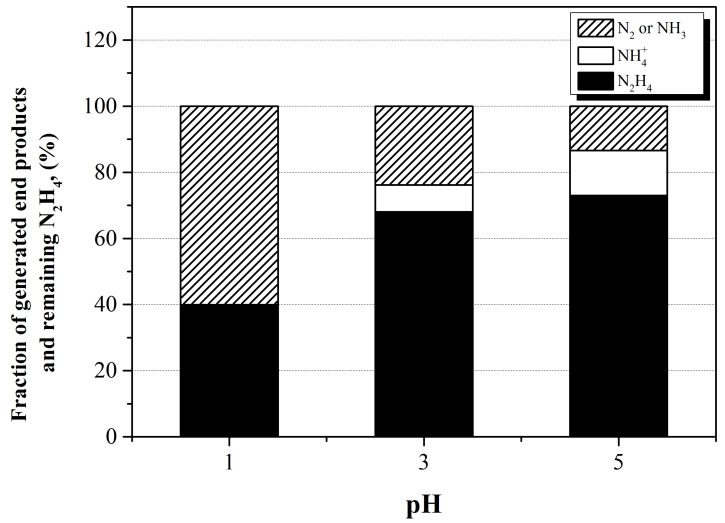
Fraction of radiolysis products and chemical species of N_2_H_4_ at different pH after 40 kGy of γ-ray irradiation.

To confirm the effect of HNO_3_ on the decomposition mechanism of N_2_H_4_ in the N_2_H_4_–Cu^+^ solution, the fraction of remaining N_2_H_4_ and end products in each sample after 40 kGy of absorbed dose irradiation at pH 1, 3, and 5 was analyzed. The initial concentrations of N_2_H_4_ in the solutions were 50 × 10^−3^ mol dm^−3^. The result is represented in Figure 7. As mentioned above, NH_3_ is converted into NH_4_^+^ ion in the solution because of the acidic condition. As shown in Figure 7, NH_4_^+^ ions were not generated after 40 kGy of absorbed dose irradiation at pH 1. Therefore, it was considered that most of N_2_H_4_ was decomposed into N_2_. The obtained result at pH 1 can be explained as follows. At pH 1, N_2_H_4_ exists in the form of N_2_H_6_^2+^ as a result of hydrolysis, as shown in Equation (3). The N_2_H_6_^2+^ ion generated N_2_ through the decomposition reaction, as indicated in Equation (5).

In addition, the amount of end products of N_2_H_4_ decomposition were decreased with increasing pH. This was the case because the large amount of H^●^ produced by the reaction between H^+^ ions and e_aq_^−^ affected the N_2_H_4_ decomposition, as shown in Figure 5. For this reason, the decrease in the concentration of end products of N_2_H_4_ decomposition following an increase in the pH was caused by a decrease in the N_2_H_4_ decomposition reaction.

However, the concentration of NH_4_^+^ ions generated after irradiation increased with increasing pH. This was the case because the N_2_H_4_ exists as a form of the N_2_H_5_^+^ ion rather than the N_2_H_6_^2+^ ion as the pH increases. As indicated in Equations (6)–(23), the end products of the reactions with a high reaction rate, among the consecutive reactions of N_2_H_5_^+^ ion decomposition in which H^●^ participated, were mainly N_2_ and NH_3_. The NH_3_ reacted with the H^+^ ion in an acidic condition and existed in the form of NH_4_^+^ ions, as mentioned above. NO_3_^−^ ions were also related to the generation of N_2_ and NH_3_. The reaction between the N_2_H_5_^+^ ion and NO_3_^●^ shown in Equation (43) might affect the generation of N_2_H_4_^●+^, NO_3_^−^ ion and H^+^ ion. As shown in Equations (15) and (16), the end products generated by the reactions of N_2_H_4_^●+^ were mostly N_2_ and NH_3_. Otherwise, the N_2_H_2_ is produced when the N_2_H_4_^●+^ reacts with NO_3_^−^ ions, as shown in Equation (42). N_2_H_2_ is the intermediate of N_2_H_4_ decomposition and it generates ^●^N_2_H_3_ after the reaction with H^●^, as represented in Equation (23). The end reaction products involving ^●^N_2_H_3_ are also N_2_ and NH_3_, as represented in Equations (17)–(22). The NH_3_ generated by the reaction between N_2_H_5_^+^ and NO_3_^●^ also existed in the form of NH_4_^+^ ion in the acidic condition. Therefore, it is concluded that HNO_3_ can affect the decomposition of N_2_H_4_ through the mechanisms listed in Equations (5)–(23) and Equations (42) and (43) by investigating the end product of the expected decomposition paths.

## 4. Materials and Methods

### 4.1. Chemicals and Sample Preparation

Hydrazine monohydrate (Junsei, Tokyo, Japan, 98.0%), nitric acid (EMSure, Darmstadt, Germany, 65.0%) and copper (I) chloride (SIGMA-ALDRICH, St. Louis, MO, USA, 97.0%) were used to prepare N_2_H_4_–Cu^+^–HNO_3_ solution in this study. The conditions of each sample is listed in Table 4. All the solutions contain 50.0 mM of N_2_H_4_. In order to investigate the effects of Cu^+^ ions on N_2_H_4_ decomposition, the solutions were prepared according to the presence of 0.5 mM of copper ions. Each sample was adjusted to pH 3 by adding nitric acid. To analyze the effects of HNO_3_ on N_2_H_4_ decomposition, each solution was adjusted to pH 1, 3, and 5 by adding 144.7 mM, 50.8 mM, and 49.9 mM of nitric acid, respectively. All the sample solutions included 0.5 mM of copper ions. The 30 mL of sample solutions were stored in the 50 mL vials. After storing the solution in the vial, the nitrogen purging was conducted for 10 min. during the γ-ray irradiation.

### 4.2. γ-rradiation

A high-dose γ-ray irradiator (Co-60 source) at the Korea Atomic Energy Research Institute was used for irradiation on the solutions. Quantities of 0, 5, 10, 20, and 40 kGy of absorbed doses were given to each sample to compare the dose effects on the decomposition of N_2_H_4_. All irradiation experiments were carried out with a dose rate of 10 kGy/hr at room temperature.

### 4.3. Analysis

The concentration of chemical species of N_2_H_4_ in the solutions was measured using a UV Spectrometer (DR 5000, Hach Co., Ames, IA, USA). The p-dimethylaminobenzaldehyde method was applied to detect the chemical species of N_2_H_4_.

## 5. Conclusions

The radiolysis of N_2_H_4_ in the N_2_H_4_–Cu^+^–HNO_3_ solution during γ-ray irradiation was verified through the irradiation experiment and the analysis of a chemical species of N_2_H_4_ concentration. When copper ions were present in the N_2_H_4_–HNO_3_ solution, the N_2_H_4_ decomposition, via the decomposition of the Cu^+^N_2_H_4_, complex was promoted by the catalytic reaction of Cu^+^ ions. HNO_3_ also accelerated the N_2_H_4_ decomposition in the N_2_H_4_–Cu^+^–HNO_3_ system through the influence of the H^+^ ion and NO_3_^−^ ion. This is because H^●^ produced by the reaction between H^+^ ion and e_aq_^−^ participated in the N_2_H_4_ decomposition reaction. Owing to the H^+^ ion effect, the N_2_H_4_ decomposition during irradiation was raised when the pH was decreased. NO_3_^−^ ion and NO_3_^●^ led to an increase in the N_2_H_4_ decomposition through the reaction with N_2_H_4_^●+^ or the reaction with N_2_H_5_^+^. These additional paths, due to the existence of the Cu^+^ and NO_3_^−^ ions, improved the N_2_H_4_ decomposition under irradiation condition. These findings can be applied, in accordance with the characteristics of radiolysis, to define the conditions of N_2_H_4_ concentration during chemical decontamination processes.

## Figures and Tables

**Table 1:** G(–N_2_H_4_) values in the N_2_H_4_–HNO_3_ and N_2_H_4_–Cu^+^–HNO_3_ solutions of which [N_2_H_4_]_0_ = 50 × 10^−3^ mol dm^−3^ at 20 kGy of absorbed dose.

Total Dose (kGy)	G(–N_2_H_4_) Value (10^−7^ mol/J)	
	w/o Cu^+^ Ions	w/Cu^+^ Ions
20	4.52	6.24

**Table 2:** G(–N_2_H_4_) values in the different pH of N_2_H_4_–Cu^+^–HNO_3_ solutions of which [N_2_H_4_]_0_ = 50 × 10^−3^ mol dm^−3^ at 40 kGy of absorbed dose.

Total Dose (kGy)	G(–N_2_H_4_) Value (10^−7^ mol/J)		
	pH 1	pH 3	pH 5
40	7.49	3.98	3.35

**Table 3:** G(–N_2_H_4_) values of N_2_H_4_–Cu^+^–HNO_3_ and N_2_H_4_–Cu^+^–H_2_SO_4_ solutions of which [N_2_H_4_]_0_ = 50 × 10^−3^ mol dm^−3^ at 40 kGy of absorbed dose.

Total Dose (kGy)	G(–N_2_H_4_) Value (10^−7^ mol/J)	
	Containing HNO_3_	Containing H_2_SO_4_
40	3.98	1.25

**Table 4:** Sample preparation.

Sample Solution	Concentration (mM)		
	N_2_H_4_	Cu^+^ Ions	HNO_3_
pH 1	50	0.5	144.7
pH 3 (without Cu^+^ ion)	50	-	50.8
pH 3 (with Cu^+^ ion)	50	0.5	50.8
pH 5	50	0.5	49.9

## Data Availability

The data presented in this study are available on request from the corresponding author.
